# Early childhood obesity: a survey of knowledge and practices of physicians from the Middle East and North Africa

**DOI:** 10.1186/s12887-017-0865-1

**Published:** 2017-04-28

**Authors:** Inge Gies, Bader AlSaleem, Beheshteh Olang, Berkouk Karima, Gamal Samy, Khaled Husain, Mahmoud Elhalik, Mohamad Miqdady, Mohamad Rawashdeh, Mohamed Salah, Nezha Mouane, Pejman Rohani, Atul Singhal, Yvan Vandenplas

**Affiliations:** 1 Department of Pediatrics, UZ Brussel, Vrije Universiteit Brussel, Brussels, Belgium; 2 Pediatric Gastroenterology, Hepatology and Nutrition Department, Children’s Hospital, King Fahad Medical City, King Saud bin Abdulaziz University for Health Sciences, Riyadh, Kingdom of Saudi Arabia; 3 Mofid Children’s Hospital, Shahid Beheshti University of Medical Sciences, Tehran, Iran; 4 Hepatology and Nutrition Society Group Member, Maillot Hospital Algiers, Algeria, Gastroenterology, Algiers, Algeria; 5 Department of Medical Childhood Studies, Institute of Postgraduate Childhood Studies, Ain Shams University, Cairo, Egypt; 6 Pediatric Gastroenterology, Hepatology and Nutrition Unit, Department of Pediatrics, Amiri Hospital, Arabian Gulf Street, Kuwait City, Kuwait; 7 Pediatric and Neonatology Department, Latifa Hospital, Dubai Health Authority, Dubai, United Arab Emirates; 8 Pediatric Gastroenterology, Hepatology and Nutrition Division, Sheikh Khalifa Medical City, Abu Dhabi, United Arab Emirates; 9 Department of Pediatrics, Jordan University Hospital, Amman, Jordan; 10 Nestlé Nutrition, Dubai, United Arab Emirates; 11 Gastroenterology Nutrition Department, Children Hospital Ibn Sina, University Mohammed V Faculty of Medicine, Rabat, Morocco; 12 The Childhood Nutrition Research Centre, Institute of Child Health, University College London, London, UK

**Keywords:** Early nutrition, Early childhood obesity, Middle East and North Africa region, Growth charts, Overweight, Professional education

## Abstract

**Background:**

Childhood obesity is one of the most serious public health issues of the twenty-first century affecting even low- and middle-income countries. Overweight and obese children are more likely to stay obese into adulthood. Due to the paucity of data on local practices, our study aimed to assess the knowledge and practices of physicians from the Middle East and North Africa region with respect to early-onset obesity.

**Methods:**

A specific questionnaire investigating the perception and knowledge on early-onset obesity was circulated to healthcare providers (general physicians, pediatricians, pediatric gastroenterologist, neonatologists) practicing in 17 Middle East and North African countries.

**Results:**

A total of 999/1051 completed forms (95% response) were evaluated. Of all respondents, 28.9% did not consistently use growth charts to monitor growth during every visit and only 25.2% and 46.6% of respondents were aware of the correct cut-off criterion for overweight and obesity, respectively. Of those surveyed, 22.3, 14.0, 36.1, 48.2, and 49.1% of respondents did not consider hypertension, type 2 diabetes, coronary heart disease, fatty liver disease, and decreased life span, respectively, to be a long-term complication of early childhood obesity. Furthermore, only 0.7% of respondents correctly answered all survey questions pertaining to knowledge of early childhood overweight and obesity.

**Conclusion:**

The survey highlights the low use of growth charts in the evaluation of early childhood growth in Middle East and North Africa region, and demonstrated poor knowledge of healthcare providers on the short- and long-term complications of early-onset obesity. This suggests a need for both continued professional education and development, and implementation of guidelines for the prevention and management of early childhood overweight and obesity.

**Electronic supplementary material:**

The online version of this article (doi:10.1186/s12887-017-0865-1) contains supplementary material, which is available to authorized users.

## Background

Childhood obesity is one of the most serious public health issues of the twenty-first century affecting even low- and middle-income countries [1]. As per WHO 2013 estimate, 42 million children under the age of 5 years are overweight or obese [1]. Overweight and obese children are more likely to stay obese into adulthood and to develop cardiovascular diseases at a younger age [1].

Epidemiologic data show that nutrition, pre- and post-natal, affects long-term health outcomes throughout life [2]. The first 1000 days of life (from conception to 2 years of age) are recognized as a critical time period when unmet nutritional needs may adversely impact short- and long-term health and physical and psycho-motor development [3]. The growth acceleration hypothesis proposes that faster post-natal growth (upward weight percentile crossing – particularly in infancy) may program several components of the metabolic syndrome, including insulin resistance, higher low-density lipoprotein cholesterol concentration, and higher blood pressure, resulting in obesity [4]. Initiation and longer duration of exclusive breastfeeding, lower protein intake during the first 2 or 3 years of life, and introduction of complementary feeding between the age of 4–6 months have all been associated with a lower risk of becoming overweight [5–11]. Nutritional approaches in the perinatal period may potentially contribute to the lifetime burden of non-communicable diseases [2] and, at present, is a major focus of nutritional research [12].

Although guidelines have been published on both the prevention and management of early childhood obesity, no studies have evaluated the knowledge and daily practice of healthcare providers. The main objective of this study was to assess the knowledge and practices of healthcare providers (general physicians, pediatricians, pediatric gastroenterologist, neonatologists) living in the Middle East and North Africa (MENA) region with respect to early-onset obesity.

## Methods

A group of national and international pediatricians specializing in pediatric obesity developed a questionnaire to investigate the attitudes of MENA physicians towards the early onset of overweight and obesity in this region (see Additional file 1 for questionnaire).

There are no universally agreed upon definitions for overweight and obesity in early childhood [13]. For the study, overweight was defined as weight-for-length above the 85th percentile and obesity was defined as weight-for-length above the 95th percentile [13].

The questionnaire was divided into two main sections. The first section included seven questions on demographic details of participants such as age, gender, practice setting, country of origin, and specialism. The second section included six questions assessing physicans’ knowledge about early childhood overweight and obesity, including the importance of early childhood obesity for short- and long-term health, systematic use of growth charts during each child’s visit, growth parameters (weight, length, head circumference, weight/height ratio, or a combination of previous parameters), cut-off criteria for infant overweight and obesity, and awareness of obesity comorbidities (cardiovascular disease, fatty liver disease, decreased life span, hypertension, type 2 diabetes).

Questionnaires were distributed between March and October 2015 to attendees of national and regional general pediatric meetings in participating countries from the MENA region. Descriptive statistics were used to summarize the demographics of the respondents and their responses to the questions.

## Results

### Physician characteristics

A total of 1051 questionnaires were retrieved between March and October 2015. Six questionnaires were excluded because they were incomplete and a further 46 were excluded because the physician’s specialty was not reported. Analysis was performed on data from 999 completed survey forms (95% response rate) (Table 1). Seventeen countries from the MENA region (Algeria, Egypt, Iran, Iraq, Jordan, KSA, Kuwait, Lebanon, Macedonia, Morocco, Oman, Palestine, Sudan, Syria, Tunis, UAE, and Yemen) participated in the survey. The largest proportion of respondents were from Morocco (21.2%), Jordan (12.4%), Iran (12.8%), KSA (11.1%), Egypt (8.4%), Algeria (8.4%), Lebanon (8.1%), Kuwait (7.5%), and UAE (7.3%).

**Table 1:** Characteristics of physicians surveyed

Characteristics	Number (N)	Percentage (%)
Total	999	100
*Age*		
< 40 years	434	43.4
40–50 years	293	29.3
50–60 years	223	22.3
> 60 years	27	2.7
Not reported	22	2.2
*Gender*		
Male	533	53.3
Female	452	45.2
Not reported	14	1.4
*Specialty*		
General physicians	222	22.2
Pediatrics	707	70.8
Pediatric gastroenterologists	58	5.8
Other (Neonatologists and NICU specialists)	12	1.2
*Practice facility*		
Government	786	78.7
Private	204	20.4
Other or not reported	9	0.9
*Country of practice*		
Algeria	84	8.4
Egypt	84	8.4
Iran	128	12.8
Jordan	124	12.4
KSA	111	11.1
Kuwait	75	7.5
Lebanon	81	8.1
Morocco	212	21.2
UAE	73	7.3
Others (Canada, Iraq, Macedonia, Oman, Palestine, Sudan, Syria, Tunis, Yemen)	27	2.7

Of the participating physicians, 54.1% of respondents were male, 78.7% worked at government facilities, and 90.5% were in full-time employment. Most respondents were pediatricians (71%) followed by family doctors (22%). The majority of respondents (74%) were aged below 50 years (44% were below 40 years, 30% were 40–50 years, 23% were 50–60 years, and 3% were over 60 years). Furthermore, only 0.7% of the surveyed physicians responded correctly to all the survey questions.

### Routine use of growth charts

Growth charts were used by more than 70% of respondents aged below 40 years and between 50 and 60 years. Growth charts were not used by 28.9% of respondents at every visit, and this finding was highly prevalent in those over 60 years (34.6%). Growth charts were more frequently used by physicians practicing in Algeria (94%), UAE (91.7%), Lebanon (89.9%), and Morocco (86.3%), than in Kuwait (45.9%) and Iran (31.5%). A higher percentage of surveyed male physicians (32.6%) did not use growth charts compared with female physicians (24.7%).

**Fig. 1:**
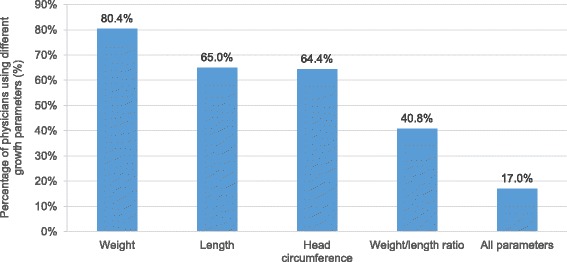
Parameters used to evaluate growth

Growth parameters such as weight, length, head circumference, and weight/length ratio were commonly assessed by physicians (Fig. 1). These findings were reflected equally across age groups. The majority of respondents across all age groups did not monitor weight/length ratio, with 64.5% of respondents aged below 40 years, 57.3% from 40 to 50 years, 53.4% from 50 to 60 years, and 51.9% above 60 years. Only 17% of all respondents monitored all parameters.

**Fig. 2:**
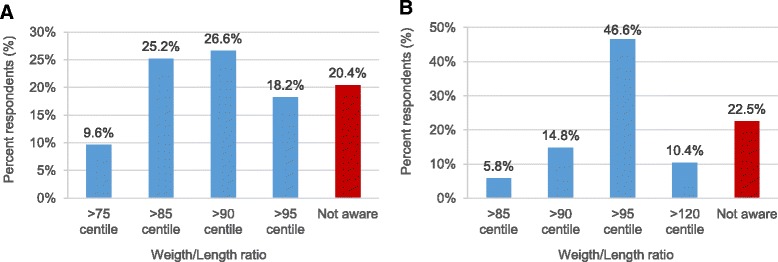
Cut-off for defining overweight (**a**) and obesity (**b**)

Cut-off for overweight was defined as weight/length ratio ≥ 85 percentile [13] by 25.2% and obesity was defined as weight/length ratio ≥ 95 percentile [13] by 46.6% of the respondents (Fig. 2). Almost 20% of physicians did not know the cut-off for overweight and obesity during infancy. Table 2 shows that the definition of overweight and obesity was similar across physicians of all age groups.

**Table 2:** Cut-off for defining overweight (A) and obesity (B) categorized by physician age

Weight/Length (percentile)	<40 years	40–50 years	50–60 years	>60 years
*[A] Defining overweight*				
*n*	*421*	*285*	*215*	*25*
>75	11.2%	9.8%	7.4%	8.0%
>85	22.1%	28.8%	24.2%	36.0%
>90	28.7%	22.5%	27.9%	28.0%
>95	16.9%	17.5%	21.4%	8.0%
Not aware	21.1%	21.4%	19.1%	20.0%
*[B] Defining obesity*				
*n*	*418*	*282*	*214*	*22*
>85	7.2%	4.3%	5.1%	9.1%
>90	16.0%	14.5%	12.1%	13.6%
>95	43.8%	51.1%	45.3%	45.5%
>120	9.1%	8.9%	15.4%	9.1%
Not aware	23.9%	21.3%	22.0%	22.7%

### Perception of early childhood obesity as a medical problem

The majority of physicians, regardless of age group, practicing in the MENA region (83.1%), thought childhood obesity was a serious issue. However, early childhood obesity was not considered a serious problem by 16.9% of respondents.

### Awareness of early childhood obesity-related comorbidities

**Fig. 3:**
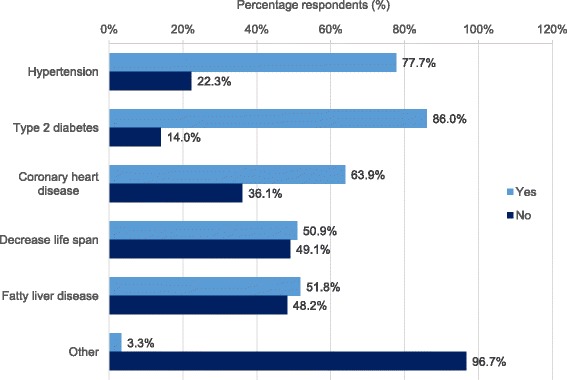
Prevalence of awareness of early childhood obesity-related comorbidities

The awareness of obesity-related comorbidities such as hypertension, type 2 diabetes mellitus, coronary heart disease, decreased life span, and fatty liver disease were assessed (Fig. 3). A combination of all listed comorbidities was only known to 32.1% of physicians. Only half of all respondents (50.9%) thought decreased life span was associated with early childhood obesity. Furthermore, most respondents (96.7%) were not aware of other obesity-related comorbidities.

## Discussion

The MENA region is known to harbor the highest prevalence of type 2 diabetes in adult populations worldwide, with underlying obesity as one of its main causes [14]. Data on early childhood obesity in the region are sparse, but clearly indicate a trend of increasing early-onset obesity [15] and increasing prevalence of metabolic syndrome in the childhood obesity population [16, 17]. Changes in dietary pattern, particularly early introduction of sweetened beverages and premature introduction of cow’s milk and complementary feeding, have been linked to early-onset obesity in the MENA region [18].

Therefore, healthcare professionals (HCPs) play a crucial role in the prevention and early diagnosis of infant and childhood obesity. Overall, our study confirms that HCPs in the MENA region regard early childhood obesity as being a serious healthcare burden. However, 16.9% of the physicians did not consider early childhood obesity as a serious condition.

Our study highlighted two key problems. Firstly, infant obesity appears to be underdiagnosed due to the lack of standardized use of growth charts during infant consultations. Importantly, one third of the questioned physicians did not use growth charts during routine consultations, and this was seen uniformly across age groups. One of the reasons for not plotting growth data could be the lack of time or the absence of knowledge on how to effectively use growth charts. This trend is not only demonstrated in our study, but has been reported by other studies [19–23] conducted worldwide. Two Australian studies revealed that about two-thirds of general practitioners do not routinely measure children’s height or weight [19, 20]. A recent study comparing the attitudes, skills, and practices in childhood obesity management of physicians practicing in Poland, Ukraine, France, and Italy found that 80% of Italian, 48.7% of French, 32.5% of Polish, and 6.1% of Ukrainian doctors routinely plotted growth charts [21]. Data from Israel and the United States suggested that some HCPs did not use growth charts because of lack of knowledge on interpretation of data [22, 23]. This was confirmed by our survey results because a varied response to cut-off values for overweight and obesity was recorded. A reason for this may due to the lack of international uniformity in cut-off values for the diagnosis of childhood obesity, debating the use a global or local growth charts, and which age groups to monitor regularly. No consensus exists on the definition of infant obesity, underlying the urgent need for standardization and simplification of definitions in infant and childhood obesity.

Secondly, physicians from the MENA region appear to not be aware of the complications associated with early-onset obesity. In our study, 22.3, 14, 36.1, and 48.2% of HCPs did not correlate hypertension, type 2 diabetes, coronary heart disease, and fatty liver disease, respectively, to be a long-term complication of childhood obesity. Most strikingly, almost half of all respondents (49.1%) did not associate decreased life span with childhood obesity. Data from Mazur et al. [21] stressed that most primary healthcare providers recognized the need for professional education in obesity management and the need for training in dietary counseling. Furthermore, Walker et al. [24] and Crawford et al. [25] reported that lack of training is a barrier to proper management of obesity, and that regular and repeated training of both HCPs and the general community is needed to raise and maintain the awareness of the complications of childhood obesity. Furthermore, only 0.7% of physicians responded correctly to all the survey questions, warranting the need for repeated professional education, to leverage expertise and improve overall health outcomes within the MENA region.

The main limitation of our study is that the information was primarily based on feedback from respondents practicing in nine out of the 17 participating MENA countries, which may not be representative of the overall situation. Furthermore, because physicians who completed the questionnaire were self-selecting (i.e. causing a bias due to individuals selecting themselves into a group), we cannot be sure that respondents were representative of the physicians in their country or region. This is particularly important because of the large and diverse physician population in this region. We used descriptive statistics to analyze survey data as only 0.7% of physicians responded correctly to all the survey questions. We have not used inferential statistical models, as they would not provide additional information to the already evident need for repeated professional education in the MENA region.

## Conclusions

This study was the first to report practices and knowledge on use and interpretation of growth charts and on the knowledge of early childhood obesity of HCPs working in the MENA region. Since this region in prone to a high incidence of (childhood) obesity, our data could improve strategies for proper recognition and diagnosis of early-onset obesity, and further stress the need for repeated education of physicians on childhood obesity. Data from this study may be used to develop best practice recommendations that can be easily adapted to a local setting with the final aim of decreasing the incidence of early-onset obesity and reducing the long-term burden on the healthcare system.

Use of growth charts in the evaluation of childhood growth is low in the MENA region, which is similar to Western countries. Knowledge on the short- and long-term complications of early-onset obesity is limited in this region, and the development and implementation of clear guidelines on the prevention and management of childhood obesity are warranted.

## Additional file

Additional file 1: Description of data: English version of the questionnaire used in the study. (DOCX 30 kb)

## Abbreviations

BF: Breastfeeding
BMI: Body Mass Index
CF: Complimentary Feeding
HCP: Health Care Professional
HM: Human Milk
MENA: Middle East and North Africa
WHO: World Health Organization
