# Supplementary material for: Early childhood obesity: a survey of knowledge and practices of physicians from the Middle East and North Africa
Source: BMC Pediatr. 2017 Apr 28;17:115. doi: 10.1186/s12887-017-0865-1 (PMC5408831; doi:10.1186/s12887-017-0865-1)
Supplement: Additional file 1: — Description of data: English version of the questionnaire used in the study. (DOCX 30 kb) [file 12887_2017_865_MOESM1_ESM.docx]

**Supplementary Material – Questionnaire**

Dear colleague,

Greetings,

In an effort to better understand obesity in the first 2 years of life in the region; we would appreciate your response to the below questionnaire. This information will be used strictly for research purposes.

**Part I**

1. What’s your specialty?
   1. General physician
   2. Pediatrics
   3. Pediatric gastroenterologist
   4. Family physician
   5. Other, please specify ………………………..
2. Do you practice in:
   1. Government facility
   2. Private facility
3. Do you practice in:
   1. Clinic
   2. Hospital
   3. Other, please specify ………………………..
4. What’s the name of the country/city where you practice? ……………..……………./………………………….
5. Gender:

a) Male b) Female

1. Age group:

a) <40 years b) 40–50 years c) 50–60 years d) >60 years

1. Do you work:

a) Full time b) Part time

**Part II**

1. How serious do you think obesity is in the first 2 years of life in your country?
   1. Very serious
   2. Serious
   3. Not serious
2. In your daily practice, is it practical to plot growth parameters for every child at every visit?
   1. Yes
   2. No
3. Which parameters do you use in evaluating infant growth (up to 2 years) in your practice? (you can give more than one answer)
   1. Weight
   2. Length
   3. Head circumference
   4. Weight/Length
4. What are your cut-off points for diagnosing overweight in the first 2 years of life?
   1. Weight/Length >75 centile
   2. Weight/Length >85 centile
   3. Weight/Length >90 centile
   4. Weight/Length >95 centile
   5. I am not aware of cut-off points
5. What are your cut-off points for diagnosing obesity in the first 2 years of life?
   1. Weight/Length >85 centile
   2. Weight/Length >90 centile
   3. Weight/Length >95 centile
   4. Weight/Length >120 centile
   5. I am not aware of cut-off points
6. Which of the following do you consider as potential long-term complications of obesity in the first 2 years of life? (you can give more than one answer)
   1. Hypertension
   2. Type 2 diabetes
   3. Coronary heart disease
   4. Decreased lifespan
   5. Fatty liver disease
   6. Other, please specify ………………………..
